# Bilateral Subdural Hematoma following Ventriculoperitoneal Shunt Insertion in a Ten-month Old Tanzanian Female with Congenital Hydrocephalus: An Uncommon Presentation

**DOI:** 10.24248/eahrj.v5i1.646

**Published:** 2021-06-11

**Authors:** Jay Lodhia, Sakina Mehboob Rashid, Abdallah Msemo, Rune Philemon, Adnan Sadiq, Kondo Chilonga, David Msuya

**Affiliations:** a Department of General Surgery, Kilimanjaro Christian Medical Center, Moshi-Tanzania; b Kilimanjaro Christian Medical University College, Moshi-Tanzania; c Department of Pediatrics and Child Health, Kilimanjaro Christian Medical Center, Moshi-Tanzania; d Department of Radiology, Kilimanjaro Christian Medical Center, Moshi-Tanzania

## Abstract

There is an unmet need for the treatment of hydrocephalus in Tanzania. Thousands of newborns each year in the region are affected by this condition and access to care remains a challenge. While treatment options like cerebrospinal fluid diversion through ventriculo-peritoneal shunting are within the skill set of general surgeons, the potential complications represent an additional challenge. We present a 10-month-old Tanzanian female who developed bilateral-subdural hematomas after insertion of a ventriculoperitoneal shunt.

## INTRODUCTION

It is estimated that more than 100,000 newborns each year in Sub-Saharan Africa (SSA) are affected by hydrocephalus.^1^ Various methods of managing this condition are in practice worldwide, the most common ones being insertion of a Ventriculo-Peritoneal (VP) shunt and Endoscopic Third Ventriculostomy with Choroid Plexus Cauterisation (ETV/CPC).

Our centre - Kilimanajro Christian Medical Centre (KCMC) situated in Northern Tanzania provides a surgical service run entirely by general surgeons. Surgical Intensive Care Services (SICU) are capped at 8 beds and usually run at capacity thus several ICU candidates are often rejected due to lack of space.

Patients presenting with Congenital Hydrocephalus are exclusively managed by insertion of a VP shunt. While it is a relatively simple procedure, failure rates within the first year of up to 25% have been reported.^2^ Shunt failure is a life-threatening emergency and thus access to emergency neurosurgical care is very vital.

The collection of a subdural hematoma following drainage of Cerebro-Spinal Fluid (CSF) via VP shunt insertions has been studied mainly in adult patients presenting with normal pressure hydrocephalus.^3,4^ It's occurrence in SSA following VP shunt insertions in paediatric populations is unreported to the best of our knowledge. We present a case report of a 10-month-old Tanzanian female who presented with a bilateral subdural collections 2 months after insertion of a VP shunt system.

## CASE PRESENTATION

### History

A 10-month-old Tanzanian female presented to our centre with a 1-week history of progressively worsening bulging of her anterior fontanelle. Her past medical history was suggestive of congenital hydro-cephalus and insertion of a VP shunt when the child was 8 months old; 2 months prior to the current presentation. Before the shunt insertion, she had a head circumference of 51 centimetres (above the 97th percentile for her age^5^) and a ventricular index of 42%. The mother reported that despite regular pumping of the shunt, the bulging did not subside. She denied any history of convulsions, vomiting, high-pitched cry or fevers.

The child's history was otherwise unremarkable; she was born at term by normal vaginal delivery, her immunisations were up-to-date with Tanzania's immunisation guidelines and she was exclusively breastfed until the age of 3 months and then supplemented.

### Examination

Upon initial examination, an active child was observed with hemodynamically stable vitals. The anterior and posterior fontanelles were tense and bulging, with the edges of the sagittal sutures wide apart. The occipitofrontal circumference was 52 centimetres-(above the 97^th^ percentile for her age^5^). There was no neck stiffness, no opisthotonos posturing and the tone of the limbs was normal. Sunset phenomenon of the eyes was not observed and the cardiovascular, abdominal, musculoskeletal and genitourinary examination was otherwise normal.

### Diagnosis

The working diagnosis was a blocked VP shunt manifesting with bulging of the fontanelles. The physical examination did not provide clues for the definitive cause of the shunt blockage.

### Laboratory and Radiology investigations

**FIGURE 1:**
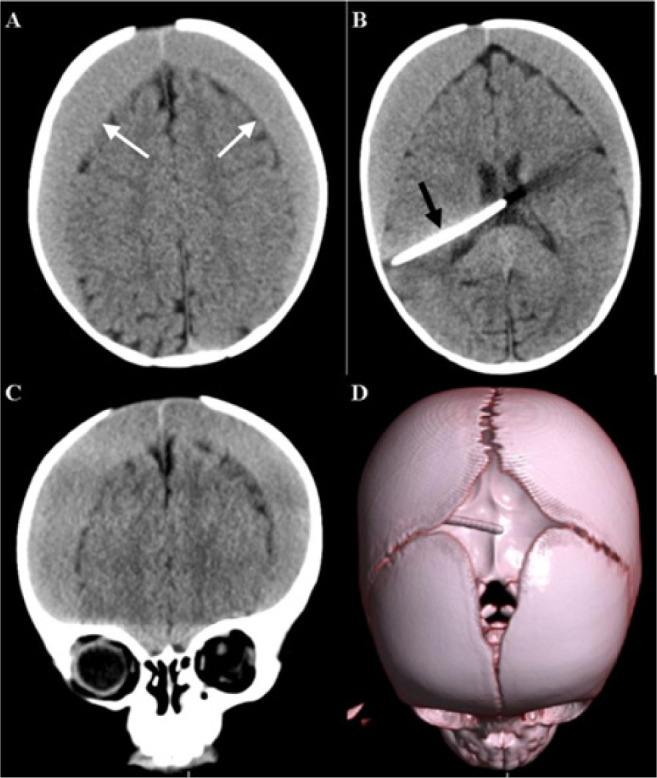
CT Scan Images A: Bilateral subdural hematoma (white arrows) B: VP shunt in situ (black arrow) C: Coronal view D: 3D reconstruction showing open fontanelles and widened sagittal suture

A complete blood count demonstrated normal parameters. A Computer Tomography (CT) scan of the head demonstrated a bilateral acute-on-chronic subdural hematoma with the shunt system in situ (Figure 1).

### Management and Clinical Course

The child was initiated on acetazolamide and planned for bilateral decompression burr hole surgery to allow for drainage of the hematoma. Intraoperatively, one burr hole was made on each side and a total of 200 millilitres of haemolysed blood was evacuated. The intraoperative-course was stable and the child recovered well from anaesthesia with neurological examination post-surgery conforming with pre-operative findings. The child was nursed in General Surgical Ward with a stable progress over 48 hours post-surgery. We would ideally have preferred post-op care in a High Care Unit. However, such services are not available at our hospital due to limited resources.

Unfortunately, on day 3, there was a sudden deterioration of the child's status while in the ward from an undetermined cause. Cardiopulmonary resuscitation was immediately initiated by the General Surgical Ward team, however it was unsuccessful and the child succumbed.

The child's rapid deterioration may have been due to various conditions. Possibilities considered by the treating team were; raised intracranial pressures secondary to subdural recollections with subsequent herniation or a seizure complicated by aspiration and hypoxia. Due to the family's preferences, a post-mortem was not conducted and an exact cause of death could not be identified.

## DISCUSSION

We present a 10-month-old child with congenital hydro-cephalus who developed a relatively uncommon complication following insertion of a VP shunt.

VP shunting is a procedure for diverting Cerebro-Spinal Fluid (CSF) to a compartment in the body which has absorptive capacity. It involves subcutaneous tunnelling of a catheter from the scalp to the abdomen in a sterile fashion, securing the proximal tip to a ventricular catheter and burying the distal tip under direct vision in the peritoneal cavity. The CSF flow towards the distal end is absorbed in the distal cavity thus resulting in lowered intracranial pressure with values closer to normal.

In patients with idiopathic normal pressure hydrocephalus, it is proposed that the collection of subdural blood is most likely due to the sudden decrease in intracranial pressure following drainage which allows the brain matter to fall away from the calvarium.^6^ It is a complication linked to high opening pressures of the CSF likely leading to drainage of large volumes of CSF during shunt insertion and subsequent high-volume drainage.^6^

Studies in high and middle-income countries have reported variable rates (11 to 25%) of shunt complications during the first year post-surgery.^2,7^ There are no exact figures describing the burden of congenital hydrocephalus and associated treatment complications within SSA. It is largely from data extrapolated from centres within the region managing this condition that estimates have been put forward.^8^

Warf et al.'s work defined the economic burden of congenital hydrocephalus in infants in SSA; they determined that the long-term benefit of managing hydrocephalus in infants for one-year can amount up to $56 billion.^9^ These figures highlight the need to manage this condition in a timely and appropriate manner while also anticipating possible complications.

Various complications following VP shunting are reported in literature including shunt obstruction, shunt migration, bowel perforation, abdominal pseudocyst formation and infection.^10,11^ Subdural hematoma collection following shunt insertion has largely been reported in patients who Various complications following VP shunting are reported in literature including shunt obstruction, shunt migration, bowel perforation, abdominal pseudocyst formation and infection.^10,11^ Subdural hematoma collection following shunt insertion has largely been reported in patients who were shunted for idiopathic normal pressure hydrocephalus with an incidence of 10% over 12 years in a Swedish study.^3^

Illingworth's series of patients who developed subdural hematomas after drainage of a hydrocephalus had all been managed by a ventriculocaval shunt.^12^ Subdural hematoma collection following ventriculoperitoneal shunting for congenital hydrocephalus has not been commonly reported in literature.

The lack of neurosurgical services at our centre - the Northern Zone Referral Hospital in Tanzania - has required general surgeons to develop proficiency in the insertion of VP shunts for patients with hydrocephalus. Investment in neurosurgical services and expertise in our region will contribute towards lessening the incidence of complications in patients post VP shunt placement. Currently, at our centre, this condition is managed entirely by general surgeons and it is the most common procedure in children under 6 months of age. Before discharge from hospital care, mothers and caregivers are counselled on the signs of increased intra-cranial pressure as well as techniques of pumping the shunt system to allow for adequate drainage.

It is clear that the allocation of human and financial capital to the health care systems in SSA is required for addressing the burden of hydrocephalus in the paediatric population and investing in access to neurosurgery care for these patients.

## CONCLUSION

Infant hydrocephalus in Tanzania represents a major Public Health challenge. The gap in adequate short and long-term post-operative care and follow-up also needs to be addressed to minimise morbidity and mortality. There is an un-met need for care resulting in immense loss of Disability Adjusted Life Years. While management is possible even by personnel who are not specialised in neurosurgery, the possibility of complications following VP shunt insertion should always be acknowledged.
